# Forty-five-year evolution of probiotic therapy

**DOI:** 10.15698/mic2019.04.673

**Published:** 2019-04-01

**Authors:** Scarlett Puebla-Barragan, Gregor Reid

**Affiliations:** 1 Departments of Microbiology & Immunology, and Surgery, Western University.; 2 Lawson Health Research Institute.

**Keywords:** probiotics, Lactobacillus, urogenital, cardiovascular, gastrointestinal, therapy

## Abstract

In the past forty-five years, the field of probiotics has grown from a handful of laboratory studies and clinical ideas into a legitimate research and translational entity conferring multiple benefits to humans around the world. This has been founded upon three principles: (i) the need for alterna-tives to drugs that either have sub-optimal efficacy or severe adverse effects; (ii) a growing interest in natural products and microbes, in particular cata-lyzed by studies showing the extent of microbes within humans and on our planet; and (iii) evidence on the genetics and metabolic properties of probi-otic strains, and clinical studies showing their effectiveness. While some man-ufacturers have sadly taken advantage of the market growth to sell supple-ments and foods they term probiotic, without the necessary human study evidence, there are more and more companies basing their formulations on science. Adherence to the definition of what constitutes a probiotic, conclu-sions based on tested products not generalizations of the whole field, and applications emanating from microbiome research identifying new strains that provide benefits, will make the next forty-five years significantly changed approaches to health management. Exciting applications will emerge for car-diovascular, urogenital, respiratory, brain, digestive and skin health, detoxifi-cation, as well as usage across the world's ecosystems.

## INTRODUCTION

The emergence of a new field of science is exciting yet invariably faces challenges to its validity and acceptance of its scope of influence. This is certainly the case for probiotics, now defined as “Live microorganisms, that when administered in adequate amounts, confer a health benefit on the host” [1].

**Figure 1:**
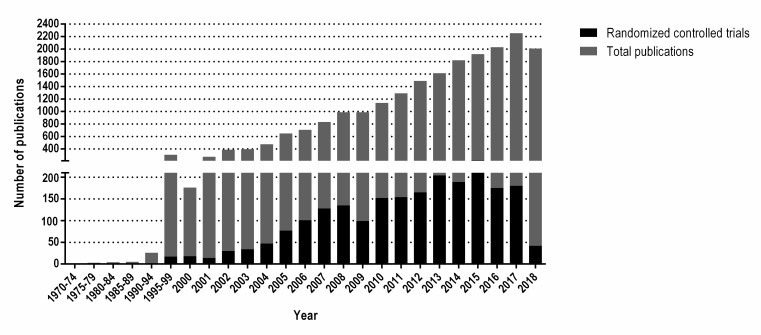
FIGURE 1: Total number of publications about probiotics on the scientific database PubMed. Gray corresponds to the total number of publications, while those corresponding to randomized controlled trials are shown in black. The Y axis was divided in two segments for ease of visualization.

The information presented in the following review has been selected with the objective of examining some key elements of probiotics, which have played an important role in the rising of the number of publications on the topic from a handful to around 20,000 on PubMed since 1973, from which over 2,000 correspond to randomized controlled trials. A detail of the number of publications per year can be visualized in **Figure 1**.

## THE MODERN-DAY ORIGINS OF PROBIOTICS

The first observations of beneficial bacteria were made by Elie Metchnikoff in 1905, when he proposed that the reason behind increased longevity in the Bulgarian population was due to the lactobacilli used to produce a yogurt commonly consumed in that region, and not the product itself as it was previously believed [2]. Nonetheless, although these remarks set the grounds for research on potential beneficial microorganisms, it was not until several decades later that formal research of probiotics begun.

Clinical observations by urologist Andrew Bruce in 1973, set the wheels in motion for considering lactobacilli as probiotics for the urogenital tract of women [3, 4]. While the rest of the field was trying to develop vaccines and therapies against uropathogenic *Escherichia coli*, none of which have so far borne fruit, he believed that replenishment of lactobacilli into the vagina where *E. coli* were dominant after repeated urinary tract infections (UTI) and antibiotic treatments, might restore homeostasis and protect the host. He also wanted to apply the same idea to patients with ileal conduits, where establishment of a ‘normal' microbiota appeared to reduce infection rates [5]. The latter never materialized but may be worth testing now with fecal microbiota transplant.

During the same era of 1960s-early 70s, Dwayne Savage and others were performing studies that showed the enormity and complexity of the intestinal microbiota in healthy subjects [3]. This was preceded by others reporting the diversity of microbes in the oral cavity [6].

The new interest in a limited microbial ecology discipline, seeded by the aforementioned studies, along with the introduction of the term ‘microbiome' by Whipps *et al.* in 1988 [7, 8], set the grounds from which the Human Microbiome Project (HMP) emerged.

Although before the early 2000's most of the microbiology studies related to humans were interested in pathogenic organisms, reviews by David Relman and Stanley Falkow [9, 10] stated the importance on paying attention to the endogenous microbes of the human body, as they could be determinant actors in health and disease. Furthermore, while the human genome project was being carried out, Julian Davies suggested that for it to be sufficiently relevant it was important to also understand the relationship between humans and the microorganisms that inhabit them [11]. The result was the HMP and Metahit Consortium that compiled an inventory of microbes in the mouth, gut, vagina, and skin of a group of humans [12, 13].

In 2001 Joshua Lederberg described the ‘microbiome' as “the ecological community of commensal, symbiotic, and pathogenic microorganisms that literally share our body space and have been all but ignored as determinants of health and disease” [14]. Early interest on the microbiome led to an increased interest on performing a large-scale investigation on the human intestinal microbiome [12]. Consequently, in November 2005, an international meeting took place in Paris, were it was recommended that a Human Intestinal Metagenome Initiative (HIMI) should be started to better understand the role of the human intestinal microbiome [12]. Furthermore, with views of aiding in the accomplishment of the objectives of the HIMI, the formation of an International Metagenome Consortium was also recommended. This meeting initiated international collaboration to elucidate and better understand the relevance of the human microbiome.

In 2008, the HMP was funded as a NIH-sponsored initiative [12]. This project was created with a vision of using high-throughput technologies to characterise the human microbiome by analyzing samples from 300 healthy volunteers at 18 different body sites, as well to also understand the role of the microbiome in health and disease [15–17]. It was also expected that the knowledge obtained would provide a standardized data resource and new technology that would help to enhance the progression in this area of study, as well as to demonstrate the relevance of the understanding and manipulation of the microbiome as a tool to improve human health. The results from this project have been the isolation and sequencing of nearly 1,300 reference strains isolated from the human body [15, 16].

The focus on beneficial microbes as distinct from pathogenic ones was all but ignored in the 1960s to early 2000s, and essentially deemed of interest only to microbial ecologists. Indeed, the President of the American Society of Microbiology even referred to probiotics as ‘snake oil' sold from the back of covered wagons [18]. Such ignorance reflects on the person making the statement rather than the progress being made in the field, but it illustrates the challenges of acceptance. Criticism continues to this day, with researchers choosing to target probiotics under the illusion of them causing widespread harm and not being proven to be safe [19], when the evidence completely contradicts such views, and indeed probiotics are effectively used to offset drug side effects [20, 21].

The updated definition of probiotics, introduced in 2001 [22] and reaffirmed in 2014 [1], along with the establishment of the International Scientific Association for Probiotics and Prebiotics (ISAPP) in 2002 [23] were major factors in stimulating research in this area and emphasizing the importance of scientific rigour and production standards for probiotics. The growth of probiotic peer-reviewed publications to around 20,000 on the scientific search engine PubMed from just over 1,000 in 2002 reflects this impact (see **Figure 1**), this tool allows the user to search publications from several scientific life-sciences and medical databases. The breadth of the definition was intentional to allow capture of a range of host benefits. Subsequently, a range of terms have been used in the literature from psychobiotics, post-biotics, next-generation probiotics to baby-biotics, notably none adequately defined and none sufficiently different that they would fall outside the existing probiotic definition. These terms seem to group probiotics in very specific clusters, with very definite uses, when in reality, most of the probiotic strains available will have more than one targeted benefit on the host. Therefore, such terminology is confusing to healthcare providers, producers and consumers. If someone truly wants to develop new terminology, they need to define the term and its scope, and show how it should be interpreted, then explore with experts in the field whether it is applicable and is likely to be accepted by the wider community.

## THE PRINCIPLES OF PROBIOTIC THERAPY

While credit is given to Elie Metchnikoff [2] for aligning fermented foods with longevity, and therefore stimulating the idea of developing such foods, the modern-day probiotics were designed for two basic reasons.

Firstly, probiotics are used to replenish organisms that are naturally in a given niche but whose numbers have been depleted and illness has occurred. This could be the use of *Lactobacillus crispatus* in the vagina to counter bacterial vaginosis or ascension of *E. coli* into the bladder [24].

Secondly, probiotic strains are selected because they have properties that counter pathogens/conditions causing illness, with an aim of restoring health and ideally allowing the indigenous beneficial microbes to return. The example would be to orally administer *Lactobacillus rhamnosus* GR-1 and *Lactobacillus reuteri* RC-14, which are not species highly prevalent in the vagina, but whose administration results in recovery from infection and the return of indigenous *L. crispatus* and *L. iners* [25].

These approaches essentially manipulate the existing microbiome, even though the term ‘microbiome' was not yet created when the probiotic applications were first conceived. Critics have argued that probiotics do not alter the gut microbiome, leading to some people then regarding them as a waste of money [26]. However, this shows a lack of understanding of the field on several counts. The 16S rRNA methods used to determine alterations in microbial abundance in the gut are far from ideal, and certainly not sensitive enough to verify that no changes are induced by probiotic intake. Furthermore, it is not a prerequisite for a probiotic to confer benefits by having to significantly change the host's gut microbiota. Rather, the health benefit can be accrued through metabolites produced by the probiotic strains as they pass through the intestine [27], and by interactions with the host's own metabolism [28] and immune system even in healthy adults [29].

## WHERE HAVE THE EARLY STUDIES TAKEN US?

By 1997, nearly a hundred studies had been carried out on probiotics for the treatment of infections to warrant a literature review. Although the authors used the term pharmaceutical probiotics, this simply reflected their use to treat disease and the antiquated categorization of only drugs being able to make that claim. They concluded that probiotics could be used to treat and prevent infectious diseases [30]. Twenty years later and this application of probiotics against infectious diseases has expanded [31], and yet the use of these products for this purpose is sporadic at best. This is in large part to the reticence of medical practitioners accepting and implementing health interventions, especially those not regulated as drugs [32]. This seventeen-year gap seems hard to accept, especially when respected groups of peers have advocated the use of probiotics, particularly for digestive function and disease [33–35].

Undoubtedly, the most extensively researched application for probiotics is to promote gastrointestinal (GI) health. Although it has been commonly believed that the GI tract is sterile *in utero*, and therefore that its colonization does not occur until birth [36], recent studies have found that the placenta, amniotic fluid, and the umbilical cord harbour microorganisms [17, 37–40]. These findings, as well as the fact that the meconium (first infant stool) is not sterile, provide a rationale to believe that the human GI tract begins its colonization during fetal development [17, 41, 42]. However, the presence of a fetal microbiota has been fiercely contested [43, 44]. Nonetheless, most of the GI colonization happens postpartum [45]. As humans develop, so does their gut microbiome, which is influenced by factors such as age, diet, stress, geography, and drug intake [17]. The human gut is a complex ecosystem with a dynamic interaction between microorganisms, nutrients, and the host [46]. Therefore, probiotic supplementation, to target and improve the gut microbiome, has been extensively researched and found to be helpful in several GI conditions.

An example is the use of probiotics to improve gut function and maturity of neonates [17]. For instance, a systematic review of 25 randomized controlled trials (RCT; *n* = 4527) where probiotics were administered to preterm (gestation <37 weeks) or low-birth-weight (<2500 g) neonates, showed benefits of probiotic supplementation such as shorter full enteral feeds, improved feed tolerance, better weight gain and growth velocity, and decreased transition time from orogastric to breast feeds; no adverse effects where reported [47]. Nonetheless, more evidence is still needed to support the claim that probiotic supplementation at an early age is safe and beneficial [48].

Furthermore, one of the most thoroughly examined areas for the use of probiotics, is the prevention of antibiotic-associated diarrhea (AAD), nonetheless it is important to note that not all probiotic strains will be as effective as others for this purpose. For instance, a recent systematic review compared the efficacy and tolerability of different probiotics for AAD, authors examined 51 RCTs (*n* = 9,569) and found that management with *Lactobacillus rhamnosus* GG (LGG) was significantly superior that with any other strain of probiotics when used for preventing the condition. While in terms of reducing *Clostridium difficile* infection rate, *Lactobacillus casei* had higher efficacy [49]. Therefore, the type of probiotic to be used should always depend on the situation of the patient, as well as the desired outcome.

Another GI condition where probiotics have been proven to be helpful is *Helicobacter pylori* colonization, which is a problem for approximately 50% of the world population [50]. There is evidence that they can be helpful in the eradication of *H. pylori* by inhibiting its growth. A meta-analysis of 14 RCTs (*n* = 1,671) found that there is a substantial 83.6% eradication rate of the pathogen with probiotic treatment vs. 74.9% with conventional therapy [33]. Additionally, probiotics significantly reduced the side effects of conventional therapy when used as a conjoint treatment. In terms of recommended strains for this condition, *Lactobacillus reuteri* DSM 17938 [51,52] and LGG [53] have been found to be effective in combination with conventional therapy.

Remarkably, the Working Group on Probiotics of the European Society of Pediatric Gastroenterology, Hepatology, and Nutrition, with the objective of providing evidence-based recommendations, published a document that reviewed existent RCTs and systematic reviews on the use of probiotics for the prevention of AAD in children [33]. Based on their results, they strongly recommend the use of either LGG or *Saccharomyces boulardii* for the prevention of the condition in children. Meanwhile, in terms of preventing *Clostridium difficile*-associated diarrhea, the Working Group recommends the use of *S. boulardii*, although with the caveat that the quality of evidence is still limited and requires further investigation. Interestingly, these recommendations are in line with those proposed for children of the Asia-Pacific region were, in addition to LGG and *S. boulardii*, *L. reuteri* was also suggested for the management of infantile colic and as a conjoint treatment with other probiotics for the management of *H. pylori* [34].

Inflammatory bowel disease (IBD) is a chronic condition of the large intestine that includes ulcerative colitis (UC) and Crohn's disease (CD). Several studies have investigated the potential benefits of administration of probiotics to patients with IBD. A meta-analysis that reviewed 22 RCTs that recruited adults with either UC or CD, with the objective of comparing probiotics to the standard treatment of 5-aminosalicylates (5-ASAs), and placebo [54] The probiotic VSL#3 was stated to be effective in inducing remission in active UC. However, for management of CD, the benefits and efficacy of probiotics are not well established, and further research is required before they could be recommended for primary care.

Not all studies of probiotics have end points for GI conditions. One highly cited example is a study published in 2001 by Finnish researchers on atopic dermatitis (AD) [55], a chronic inflammatory disease of the skin which has become a major public-health problem [56]. It is estimated to affect 15-20% of children and 1-3% of adults worldwide, and its prevalence has increased by 2-3 fold recently (particularly in low-income and industrialized countries) [57, 58].

The immune mechanisms associated with AD are characterized by a biphasic inflammation, meaning that at an initial and acute phase there is a predominant Th2-biased immune response, while chronic lesions are characterised by a Th1/Th dominance [58, 59]. Regulatory T cells as well as the innate immune system are altered in the skin of patients with AD [60]. The link between childhood impaired immune system development and an altered intestinal microbiome increasing the susceptibility to allergic and autoimmune disease [61], led to a study by Majamaa and Isolauri which found that probiotic LGG promoted local-antigen specific immune responses that prevented gut permeability defects [62]. The researchers then hypothesized that oral administration of probiotics could be helpful in the treatment of food allergies by diminishing intestinal inflammation. Levels of α_1_-antitrypsin decreased significantly as did the concentration of fecal tumor necrosis factor-α [63]. It was these studies that formed the basis for the randomized placebo-controlled trial in which LGG was administered prenatally to mothers with at least one first-degree relative (or partner) with atopic eczema, allergic rhinitis, or asthma, and to their infants for 6 months after birth. Their findings showed a significant decrease in the frequency of AD in the group that consumed probiotics vs the placebo [55]. The researchers followed the children for a further four years and reported there was still a reduced risk of AD [64]. Although this study stimulated much interest in the use of probiotics in infants and pregnant women, as well as to aid in the modulation of immune diseases, subsequent studies have varied in their findings [65, 66], and the totality of evidence has been deemed insufficient to support a therapeutic claim [67]. In terms of using probiotics to prevent AD, a meta-analysis of fourteen studies demonstrated a moderate decreased incidence (RR = 0.79 [95% CI = 0.71-0.88]) [68].

Some probiotic strains appear to confer benefits as adjuvant therapy for the treatment of adults [69]. However, a recent meta-analysis of thirteen studies aimed at using probiotic strains to treat children with AD, concluded that the evidence was not sufficiently robust for the category in general [70].

Another major area of interest in probiotic research is their use to manage urogenital tract conditions, mostly because of the high prevalence of UTI and bacterial vaginosis (BV), and the lack of effective treatment options [71,72]. A number of probiotic strains have been developed to prevent urogenital infections, *Lactobacillus acidophilus* A-212*, Lactobacillus rhamnosus* A-119*, with Streptococcus thermophilus* A-336*; Lactobacillus rhamnosus* PBO1 with *Lactobacillus gasseri* EN-153471 (EB01); and *Lactobacillus rhamnosus* Lcr35 in vaginal ovules. Strains *L. rhamnosus* GR-1 and *L. reuteri* RC-14 are the only ones approved for oral use in Canada and the United States. The positive early clinical studies performed with GR-1 and RC-14 showing improved vaginal microbiota and reduced infection recurrence, as reviewed elsewhere [73] have been confirmed by others [74–76]. The mechanisms of action include an increased ascension of probiotic and indigenous lactobacilli from rectal skin to the vagina, reduced pathogens ascension, plus localized inhibition and displacement of pathogens and priming of antimicrobial defenses [77, 78]. Anti-fungal effects have also been reported [79, 80], coinciding with improved curing of vulvovaginal candidiasis [81].

The vaginal administration of probiotic *Lactobacillus* using suppositories pioneered in the late 1980s [4], has since led to other strains being tested for urogenital health including *Lactobacillus crispatus* CTV05 to prevent recurrence of UTI [24], *Lactobacillus rhamnosus* IMC 501 with *Lactobacillus paracasei* IMC 502 [82] for vaginal health, *Lactobacillus rhamnosus* Lcr35 to aid in the management of BV and vulvovaginal candidiasis [83, 84], and *Lactobacillus gasseri* EN-153471 (EB01) to help treat BV along with antibiotics [85].

A recent study of the genome of *L. rhamnosus* GR-1 showed why it is better adapted to the vagina than Lc35 and LGG strains, by having a unique cluster for exopolysaccharide production, metabolize lactose and maltose, and better withstand oxidative stress [86].

Table 1 includes a summary of the strains with significant clinical evidence for their use as probiotics, as well as the conditions for which they have been studied.

**TABLE 1.** Probiotic strains and the condition they target.

Condition targeted*	Probiotic strains
Irritable bowel syndrome	*Bifidobacterium infantis* 35624 [122]
	*Bifidobacterium animalis* DN-173 010 [123, 124]
Constipation	*Bifidobacterium lactis* DN-173010 [125]
	*Lactobacillus reuteri* DSM 17938 [126]
AAD	Combination of *Lactobacillus acidophilus* CL 1285 + *Lactobacillus casei* LBC80R + *Lactobacillus rhamnosus* CLR2 [127, 128]
	*Lactobacillus reuteri* DSM 17938 [129]
	*Lactobacillus rhamnosus* GG [130]
	*Saccharomyces boulardii* [131]
*H. pylori* (in combination with standard therapy)	*Lactobacillus reuteri* DSM 17938 [51,52]
	*Lactobacillus rhamnosus* GG [53]
IBD	VSL#3 [54]
BV	*Lactobacillus rhamnosus* GR-1 *+ Lactobacillus reuteri* RC-14 [132,133]
	*Lactobacillus rhamnosus* Lcr35 [84, 134]
	*Lactobacillus rhamnosus* PBO1 and *Lactobacillus gasseri* EN-15347 (EB01) [85]
AD	*Lactobacillus rhamnosus* GG [55]
High cholesterol	*Lactobacillus acidophilus + Bifidobacterium lactis* [101]
	VSL#3 and *Lactobacillus plantarum* [101]

* There are online resources that provide information on probiotics tested in humans and available for purchase in the USA and Canada, with level of evidence indicated [135].

## THE ULTIMATE REBUKE OF THE SNAKE OIL MYTH

Although the level of scientific and clinical evidence for probiotics should have by now rebuked the statements from even presidents of microbiology societies [18], and the 17-year change timeframe has now passed [32], the ability to save lives should surely be the ultimate proof of validity. A recent large study from India showed such an ability in preventing sepsis in newborns [87], and the ability of numerous probiotics to prevent necrotizing enterocolitis (NEC) in low birth weight, premature babies is extremely convincing. In fact, a meta-analysis of 29 RCTs (*n =* 2,310) that looked at the effect of probiotics on experimental NEC in animal models, reached the conclusion that probiotics help to significantly reduce NEC by different mechanisms which include modulating immune function as well as by regulating inflammation, tissue injury, gut barrier and intestinal dysbiosis [88]. Furthermore, a recent meta-analysis evaluated 37 RCTs (*n* = 5,033) that looked at the effect of probiotic administration to infants of less than 37 weeks of gestation or who were born weighing less than 2.5 kg, results showed that probiotics are indeed beneficial in the prevention of severe NEC and death in preterm infants [89]. Yet, despite the evidence, and highly successful implementation in neonatal intensive care units in Canada, Australia and elsewhere, American hospitals appear reluctant to embed it into practice.

The neonatal intensive care unit is essentially a box in which the environment is mostly filled by pathogenic, drug resistant pathogens apart from the indigenous microbiota of the attendants. It is no surprise that babies requiring intubation, intravenous fluids and intensive care, and invariably given antibiotics which destroy beneficial as well as pathogenic bacteria, are at high risk of NEC. The only means of administering to them beneficial bacteria is through mother's milk, and that is dependent on whether the mother produces milk and feeds it to the baby. The immature gut and immune system place the newborn at high risk of NEC, so it is perhaps not surprising that the administration of probiotic bacteria protects against the pathogens and improves gut barrier function. The healthcare savings from preventing NEC are enormous, so it seems counter intuitive to not make probiotic therapy standard practice.

However, many clinicians are still reluctant to prescribe probiotics, perhaps due to fear and lack of knowledge about the area, which is further heightened by isolated reports of probiotic-induced sepsis [90]. Nonetheless, these cases are rare and often occur in immunocompromised patients. Also, the evidence available shows that, in most patients, the benefits outweigh the risks. In fact, a meta-analysis from 2016, found that there is a decreased incidence of culture-proven sepsis when probiotics are administered [91]. But, it is understandable, that practitioners proceed with caution when considering the administration of probiotics to high risk patients, and a guide to probiotics for this purpose has been published [20].

Despite guidelines for what constitutes a probiotic being published in 2002 [22], meta-analyses often fail to appreciate that there are strain to strain differences [92], and pool together studies performed on multiple product formulations. If a product has not been proven to benefit humans, it should not be called probiotic and therefore not included in a meta-analysis. If a probiotic for one condition fails in another, then it should not be recommended for the latter, and physicians should not then conclude that all probiotics work or don't work. In some ways it is like comparing Warfarin with Lipitor, when both drugs have different purposes, even if both might benefit heart health. If different probiotics provide benefits for the same condition, as it has been found for NEC [88, 89], then it seems reasonable to include them in a meta-analysis. However, a lack of understanding of probiotics can lead to scientific publications that make unsubstantiated conclusions and find their way to the mainstream media claiming that probiotics might not be as useful as commonly believed [93] or deeming them even potentially harmful to the host microbiota [94]. To make things even worse, when reporting these studies to the lay audience, the BBC went as far as to use a sensationalist headline, labeling probiotics as ‘quite useless', without considering that the experimental design of said studies did not provide sufficient evidence to make that sort of generalizations or any clinical claims [95]. This is like stating all drugs are quite useless. Sadly, even though renowned experts in the field [96, 97], and even the International Scientific Association for Probiotics and Prebiotics [98], made statements that pointed out the many flaws of this argument, the idea had already spread through mainstream media and the damage will be hard to revert.

## THE NEXT FORTY-FIVE YEARS

Predicting the future has never been easy and is usually based on what we know today. To that extent, it seems clear that probiotics will be used to reduce depression and anxiety [99, 100], and potentially other forms of mental illness. Certainly, trials are underway using fecal microbiota transplant for multiple sclerosis, Parkinson's disease, and dementia, so within forty-five years, the strains able to confer an effect will have been identified, tested and be part of a microbial intervention medical practice. A more profound step will be to implant probiotic strains directly into the brain, perhaps to counter disease-causing organisms, or to produce certain chemicals, such as γ-aminobutyric acid or serotonin at specific sites to try and improve function. This will require an ability to manipulate the strains and control their spread beyond the implanted site – all well within the capabilities of molecular genetics and biomedical engineering. It is unlikely that bacteriophages or organisms like *Bacillus thuringiensis* var. *israelensis* that kill mosquito larvae, will be defined as probiotics given that they don't benefit their host bacterium, but they too could be candidates for implantation into the brain, as might microbes that kill viruses. The ethical issues may take longer to unravel, depending on what metabolites are produced, and what functions they influence.

There will be no harder ethical questions raised than if probiotics influence organ development of the fetus and its life-long course. This is certainly feasible given that AD risk can already be reduced with maternal treatment. Whether the probiotics will be implanted into the uterus or their functions conveyed via the mother's metabolism remains to be seen, but either could occur. There are clearly compounds, including neurochemicals, vitamins, lipids and peptides produced by microbes that already influence organ development and function [101–105]. There is also preliminary evidence for improving cognitive function in adults [106].

The research necessary to provide accurate modulation of fetal determinants of health will require an understanding of the extent to which microbes and their metabolites, whether in the mother or uterine/placental environment, influence human physiology without inducing long term adversity. This will be difficult to achieve and will likely require sensory systems that detect changes at the sub-cellular level. Probes like the iKnife [107] could potentially be minimized further, or nano-detection systems put in place [108]. Determining the contribution of nutrients, hormones and stem cell activities will further complicate the identification of critical microbes to be administered to optimize fetal development. But studies in which probiotic strains are already being used during pregnancy will allow assessments of the newborn's organs and early year development, that will at least allow hypothesis generation, and markers to be available for testing different interventions, such as high folate producing probiotics.

Two other areas that will see significant advances in the next forty-five years are probiotics for cardiovascular management and to reduce uptake and damage caused by environmental toxins. There is already a growing literature on probiotic strains lowering cholesterol and improving blood pressure [109, 110], even meta-analyses have found a significant reduction of serum total cholesterol when administering certain probiotics (namely *L. acidophilus + B. lactis*, VSL#3, and *L. plantarum*) [101]. Once comparative studies against statins are performed, we will know the percentage risk reduction for cardiovascular disease and be able to identify patients who can benefit from the probiotics prior to needing the drugs or as an adjunct to the statins. The animal studies showing a further effect on cardiac remodeling to reduce heart failure after ischemic injury [111, 112], likely through increasing adiponectin, warrants human studies to determine if probiotic strains given right after infarction might increase longevity. Another application could be for *Bifidobacterium* strains to lower p-cresol sulfate, trimethylamine n-oxide and indole levels which are known risk factors for atherosclerosis [113, 114].

Lastly, in a world with ever-increasing environmental pollution [115], the question is not when water will become pure enough to drink, as that day will never come, but how can humans cope with the inevitable intake of toxic products? The oro-gastrointestinal microbes provide a natural barrier that could be further enhanced by probiotics. It is well known that bacteria in the intestine can increase or decrease drug toxicity and uptake [116], and examples are mounting of probiotic bacteria reducing uptake of inadvertent intake of toxic compounds [117–119]. Blocking uptake through binding to the compounds or degrading them while they are in the GI tract, will become an application of probiotics, made even more effective by matching strains with foods and water whose toxin content is known through detectors that will become widely available. The ability of consumers to extract toxins and the many pharmaceutical agents contaminating our water supply [120] at their tap, will become standard in households, but ingesting foods with microbes that are highly adapted to detoxification will further help to reduce the health effects of these chemicals.

## IN SUMMARY

The past forty-five years have seen probiotic microbes identified, tested, and applied to patients and consumers around the world. The over 40 billion-dollar market [121] reflects not only an interest in natural therapies and desire to avoid drugs that are often ineffective or with severe side-effects, but it has come about through rigorous scientific research. There may never be sufficient large, randomized placebo-controlled trials to satisfy all critics, but the numbers of lives saved and enhanced by probiotics continue to grow. As technology advances, the future will see different species applied in novel ways to further improve human well-being, and indeed the health of a range of other hosts from fish to honey bees, livestock and wildlife.

## Abbreviations:

AAD: – antibiotic associated diarrhea,
AD: – atopic dermatitis,
BV: – bacterial vaginosis,
GI: – gastrointestinal,
HMP: – Human Microbiome Project,
IBD: – inflammatory bowel disease,
LGG: – Lactobacillus rhamnosus GG,
NEC: – necrotizing enterocolitis,
RCT: – randomized controlled trial,
UTI: – urinary tract infection.
